# Snake Venom Components: Tools and Cures to Target Cardiovascular Diseases

**DOI:** 10.3390/molecules26082223

**Published:** 2021-04-12

**Authors:** Jacinthe Frangieh, Mohamad Rima, Ziad Fajloun, Daniel Henrion, Jean-Marc Sabatier, Christian Legros, César Mattei

**Affiliations:** 1 Laboratory of Applied Biotechnology (LBA3B), Azm Center for Research in Biotechnology and Its Applications, EDST, Lebanese University, Tripoli 1300, Lebanon; jacynthefrangieh@gmail.com (J.F.); ziad.fajloun@ul.edu.lb (Z.F.); 2 University Angers, INSERM U1083, CNRS UMR6015, MITOVASC, Team 2 CarMe, SFR ICAT, 49000 Angers, France; daniel.henrion@univ-angers.fr (D.H.); christian.legros@univ-angers.fr (C.L.); 3 Institut de Génétique et de Biologie Moléculaire et Cellulaire (IGBMC), INSERM, CNRS, Université de Strasbourg, 67400 Illkirch, France; mohamad.rima@hotmail.com; 4 Department of Biology, Faculty of Sciences 3, Lebanese University, Campus Michel Slayman Ras Maska, Tripoli 1352, Lebanon; 5 Faculté de Médecine Secteur Nord, 51, Institut de Neuro-Physiopathologie, Université Aix-Marseille, UMR 7051, Boulevard Pierre Dramard-CS80011, CEDEX 15, 13344 Marseille, France

**Keywords:** snake venom, cardiovascular diseases, hypotensive agent, anti-platelet agent, vasorelaxant effect, drugs discovery

## Abstract

Cardiovascular diseases (CVDs) are considered as a major cause of death worldwide. Therefore, identifying and developing therapeutic strategies to treat and reduce the prevalence of CVDs is a major medical challenge. Several drugs used for the treatment of CVDs, such as captopril, emerged from natural products, namely snake venoms. These venoms are complex mixtures of bioactive molecules, which, among other physiological networks, target the cardiovascular system, leading to them being considered in the development and design of new drugs. In this review, we describe some snake venom molecules targeting the cardiovascular system such as phospholipase A2 (PLA2), natriuretic peptides (NPs), bradykinin-potentiating peptides (BPPs), cysteine-rich secretory proteins (CRISPs), disintegrins, fibrinolytic enzymes, and three-finger toxins (3FTXs). In addition, their molecular targets, and mechanisms of action—vasorelaxation, inhibition of platelet aggregation, cardioprotective activities—are discussed. The dissection of their biological effects at the molecular scale give insights for the development of future snake venom-derived drugs.

## 1. Introduction

Snakes use their venom to neutralize and predigest prey, and to ward off or weaken predators. Several types of strategies exist [1]: (i) muscle paralysis by blocking the neuromuscular junction, (ii) alterations of the cardiovascular function to cause tissue ischemia or circulatory collapse, (iii) tissue predigestion via cell necrosis. Among venomous animals, snakes have long captured the human imagination. On the one hand, snakebite envenoming causes a serious public health issue worldwide, including a range of acute medical emergencies, with deadly consequences (WHO, 2020). The World Health Organization estimates that 81,000 to 138,000 people die every year from a snake envenomation, and 400,000 victims/year live with permanent disabilities (WHO, 2019). On the other hand, there is a growing interest in studying snake venoms for therapeutic applications. 

Venomous snakes are classified into four families (i) Atractaspididae, (ii) Elapidae, (iii) polyphyletic “Colubridae”, and (iv) Viperidae [2]. Their venom is a mixture of mostly enzymes and non-enzymatic proteins or peptides constituting 90% to 95% of the venom’s dry weight. Other components include carbohydrates, lipids, metal ions, and inorganic anions. These proteins have either (i) enzymatic activities such as metalloproteinase, serine proteinase, phospholipase A2 (PLA2), acetylcholinesterase (AChE), L-amino-acid-oxidase (LAAO), or hyaluronidase or (ii) non-enzymatic properties like natriuretic peptides, three-finger toxins, C-types lectins, proteinase inhibitors, and bradykinin-potentiating peptides [2,3,4]. Snake venom composition is not stable, and can indeed vary depending on different factors such as ontogeny, sex, snake stress, geographical distribution, and environmental and diet conditions [5]. Various studies have shown that snake venom compounds can cause a range of biological effects on the local and systemic level. The local effect is characterized by burning, bursting, or throbbing pain followed by local swelling and tissue necrosis. On the systemic level, the venom can cause different effects including neurotoxicity, myotoxicity, cardiotoxicity, nephrotoxicity, coagulopathy, and circulatory shock [6]. These life-threatening conditions caused by venom components lead scientists to study and characterize the molecules of interest with the aim of turning out these toxins into a source of life-saving therapeutics. The idea that snake venoms could be the source of remedies in various pathologies emerged from the observation of cardiovascular symptoms in humans after snake envenomation. 

Cardiovascular diseases (CVDs) can affect (i) the heart, causing conditions such as myocardial infarction, heart failure, and cardiac rhythm disturbances or arrhythmias and (ii) blood vessels through hypertension, atherosclerosis, aneurysm, and strokes. CVDs are considered the most common cause of death globally [7]; they account for 45% and 37% of all deaths in Europe and in the European Union each year, respectively [8]. The WHO described the different risk factors—including unhealthy diet, obesity, smoking, lack of physical activity, harmful consumption of alcohol, hypertension, hyperlipidaemia, and diabetes—facilitating the development of heart and vessel diseases. Among them, hypertension is a major contributor to the high prevalence of CVDs, as complications from hypertension account for 9.4 million deaths worldwide per year. For this reason, treatment of hypertension is a major issue of pharmaceutical companies in order to reduce the prevalence of CVDs. Some drug development strategies rely on natural products, such as snake venom, to treat many diseases including CVDs [9]. For example, captopril was the first drug derived from viper venom approved by the Food and Drug Administration to treat hypertension and congestive heart failure [10]. This venom-derived drug development strategy counts on the fact that snake venom compounds target ion channels (Na^+^, K^+^, and Ca^2+^ channels) and receptors (acetylcholine receptors, membrane transporters, enzymes), and consequently can partake in different physiological events. Interestingly, the biological effects of venom are due to the individual activities of their compounds or to the synergy of several molecules, therefore broadening the spectrum of their biological activities. These properties of snake venom compounds make them a valuable source for the discovery of new drugs for the treatment of several diseases including CVDs. The clinical benefits of snake venoms have emerged in two main directions related to the cardiovascular system: (i) the management of hypertension and (ii) the consequences of platelet aggregation with anti-thrombotic therapy.

In this review, we describe snake venom components that have been shown to interact with the cardiovascular system, leading to their use as a potential treatment of CVDs, and emphasize their targets and mechanism of action.

## 2. Main Components of Snake Venom and their Effects on the Cardiovascular System 

**Figure 1:**
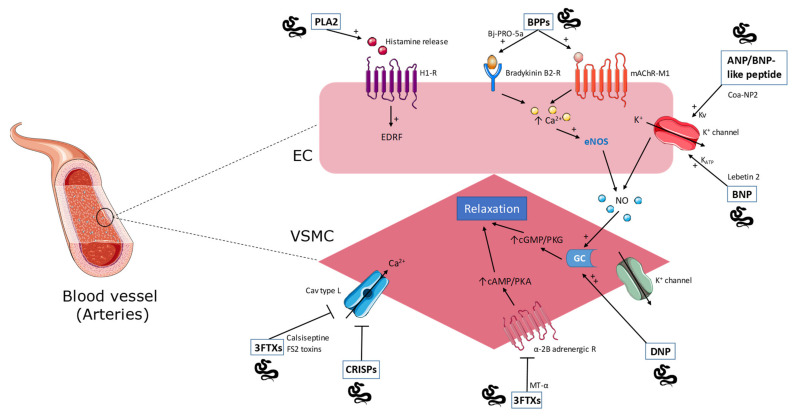
Overview of snake toxins acting on the vascular tone through activation/inhibition of receptors expressed at the surface of endothelial cells (EC) or vascular smooth muscle cells (VSMC). See text for details. PLA2: phospholipase A2; BPPs: bradykinin potentiating peptide; ANP/BNP-like peptide: atrial natriuretic peptide/brain natriuretic peptide; BNP: brain natriuretic peptide; 3FTXs: three-finger toxins; CRISPs: cysteine-rich secretory proteins; DNP: dendroaspis natriuretic peptide; MT-α: muscarinic toxin; H1-R: histamine H1 receptor; mAChR-M1: M1 muscarinic acetylcholine receptor; EDRF: endothelium derived relaxing factor; eNOS: endothelial nitric oxide synthase; NO: nitric oxide; GC: guanylate cyclase; Cav type L: L-type voltage-gated calcium channel.

**Figure 2:**
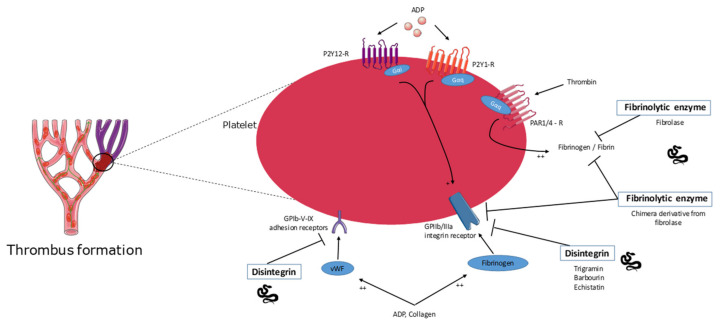
Overview of the molecular targets of snake venom toxins with antithrombotic effect. See text for details. Disintegrins and fibrinolytic enzymes, two different families of proteins, act on the surface of platelets via different receptors and glycoproteins or directly by digesting fibrin to inhibit thrombus formation. ADP: adenosine diphosphate; vWF: von Willebrand factor; GPIIb/IIIa (αIIbβ3): platelet-specific integrin receptor; GPIb-V-IX: platelet glycoprotein adhesion receptor; P2Y12-R, P2Y1-R:¨Platelet G-protein-coupled receptor for ADP; PAR1/4-R: Protease activated receptors as G-protein-coupled receptors for thrombin.

The toxins presented in this section appear in Table 1 and Figure 1 and Figure 2.

### 2.1. Phospholipase A2

Phospholipase A2 (PLA2) enzymes catalyze the hydrolysis of membrane glycerophospholipids to release lysophospholipids and arachidonic acid [11]. These enzymes, widely found in animal venoms, have diverse effects including inflammation, myotoxicity, neurotoxicity, and hypotension [12,13,14]. Among these effects caused by snake venom PLA2 (svPLA2), the hypotensive properties are of interest for the treatment of hypertension, which is an important factor of developing CVDs. As such, an in vivo study reporting the cardiovascular effects induced by snake venoms showed that two distinct phenomena appeared following venom injection: (i) a rapid decrease in blood pressure (termed cardiovascular collapse) and (ii) prolonged hypotension. In this study, PLA2 was suggested to be responsible for prolonged hypotension via vasodilatation [15]. In agreement with such observations, two PLA2 (OSC3a and OSC3b) isolated from coastal taipan (*Oxuranus scutellatus*) venom induced hypotension response without cardiovascular collapse in vivo and relaxed vascular tissue in vitro.

It was proposed that this effect involves histamine H1 receptors as the pre-administration of mepyramine, an H1 receptor antagonist, alone or in combination with heparin, an inhibitor of histamine release, attenuated the hypotensive effect of isolated PLA2 [16]. These findings are in correlation with others studies showing that svPLA2s induce histamine release by activating mast cells from different tissue, leading to increased vasodilatation and vascular permeability [17,18]. The action of histamine can be mediated through G protein-coupled receptors including H1 and H2 receptors. These two vasodilatation-mediating receptors are distributed throughout the resistance vessels in most vascular beds. H1 receptors are located mainly in endothelial cells (EC), and their activation by endogenous compounds leads to the formation of a local vasodilator substance, namely nitric oxide (NO). It spreads from EC to vascular smooth muscle cells (VSMC) to stimulate the increase of cyclic guanosine monophosphate (cGMP) level leading subsequently to the relaxation of the VSMC and the regulation of blood pressure [19]. Another PLA2 purified from the Brazilian lancehead (*Bothrops moojeni*) venom also induces an in vivo hypotension effect [20]. A crotoxin with PLA2 activity from the South American rattlesnake (*Crotalus durissus terrificus*) venom shows a vasoactive action on human umbilical vein endothelial cells suggesting an anti-atherogenic effect that is worth further investigation [21].

### 2.2. Natriuretic Peptides

Natriuresis, which is the excretion of Na^+^ ions by the kidneys, is induced by natriuretic peptides (NPs) [22], such as the endogenous atrial natriuretic peptide (ANP) brain natriuretic peptide (BNP), cardiac natriuretic peptide (CNP), and the venom originating dendroaspis natriuretic peptide (DNP). The effects of NPs are mediated through three types of receptors: natriuretic peptide receptor-A, -B, and -C (NPR-A, NPR-B, NPR-C), which play a role in the regulation of blood pressure as well as cardiovascular and renal functions [23,24]. ANP and BNP can contribute to vasorelaxation and natriuresis in animal models and humans with congestive heart failure (CHF), whereas CNP is likely to have a paracrine regulatory role on the vascular tone [25]. 

DNP has been isolated from the venom of the green mamba (*Dendroaspis angusticeps*). The biological activities of this peptide include: (i) a relaxation of aortic strips pre-contracted with KCl, (ii) stimulation of guanylate cyclase (GC) in cultured aortic myocytes and bovine aortic endothelial cells, and (iii) prevention of ^125^I-ANP binding to aortic myocytes. The vasorelaxant effect of DNP relies on its diffusion to vascular smooth muscle cells that activates GC and generates cyclic guanosine monophosphate (cGMP)-dependent protein kinase G (PKG) pathway, which, in turn, stimulates Ca^2+^ channels and ATP-sensitive K^+^-channels, hence resulting in vasodilation. The effect of DNP has been reported to be mediated through the ANP-A and ANP-C receptors and clearance receptors [26,27,28,29]. Da Silva et al. isolated and characterized a new natriuretic-like peptide known as Coa-NP2 from the Grand Canyon rattlesnake (*Crotalus oreganus abyssus*) venom that has structural homology with the ANP/BNP-like family. Coa-NP2 causes a dose-dependent hypotensive effect in rats in association with an increased NO production measured in the plasma and vasorelaxation of thoracic aortic rings. Therefore, the NO-release dependent vasodilator action of this peptide may occur by stimulation of K^+^ channels [30]. In another study, a new NP called Lebetin 2 (L2) from the blunt-nosed viper (*Macrovipera lebetina*) venom was described. The peptide showed structure similarity to BNP and was shown to have a strong cardioprotective effect in acute ischemia via the involvement of NPRs and the mitochondrial channel KATP (mitoKATP). L2 has shown an ability to (i) improve contractile recovery after regional ischemia-reperfusion, (ii) increase coronary flow and decrease severe contractile dysfunction after global ischemia, and (iii) enhance the expression of survival protein in the reperfusion myocardium as evinced by phosphorylation of signaling pathways PKCε/ERK/GSK3β and PI3K/Akt/eNOS [31]. Many other NPs from snake venoms have been identified and showed effects on the vascular system including NP2_Casca from the venom of the cascavella rattlesnake (*Crotalus durissus cascavella*) [32], PtNP-a from the eastern brown snake (*Pseudonaja textilis*), PaNP-c from the king brown snake (*Pseudechis australis*) [33], PNP from the Persian horned viper (*Pseudocerastes persicus*) [34], and three natriuretic-like peptides TNP-a, TNP-b, and TNP-c from the venom of the inland taipan (*Oxyuranus microlepidotus*) [35]. Despite the diversity of NPs isolated from snake venoms, these peptides hold the same vasorelaxant activity.

### 2.3. Bradykinin-Potentiating Peptides 

Bradykinin-potentiating peptides (BPPs) or bradykinin-potentiating factors (BPF) are known to be proline-rich oligopeptides (PROs) containing 5-14 amino-acid residues, which can be found in certain snake venoms [36]. The first BPP was extracted from the venom of the Brazilian pit viper (*Bothrops jararaca*) and shown to be an angiotensin converting enzyme (ACE) inhibitor. The peptide potentiates the hypotensive effect of bradykinin [37]. This pioneering study led to the development of captopril, the first venom-based ACE inhibitor (see Section 3.1). The decrease in blood pressure is due to two actions exerted by BPP: (i) the inhibition of the C-domain of ACE, which inhibits the formation of angiotensin II (Ang II)—a vasoconstrictor agent—from angiotensin I (Ang I), and (ii) the potentiation of the endogenous bradykinin, a vasodilator molecule, by blocking its degradation [38,39]. Morais et al. described and isolated a new member of BPPs from *Bothrops jararaca* venom. The peptide, called Bj-PRO-5a, also consists of a natural ACE inhibitor leading to vasodilation. Despite its ACE inhibitory effect, Bj-PRO-5a has been shown to promote vasodilation through the activation of muscarinic acetylcholine receptor M1 subtype (mAChR-M1) and bradykinin B2 receptor, which in turn leads to transient increases in intracellular calcium levels. The intracellular signal activates endothelial nitric oxide synthase 3 (eNOS) that induces NO production and promotes vasorelaxation [40]. Furthermore, two other BPPs from *Bothrops jararaca* venom were isolated: (i) Bj-PRO-7a, known as a mAChR-M1 agonist like Bj-PRO-5a, and (ii) Bj-PRO-10c [41]. The latter showed a significant decrease in both BP and heart rate (HR) in spontaneously hypertensive rats (SHR). It acts via the activation of GPCRs that induce a Ca^2+^i increase, glutamate, and GABA neurotransmitter release, which act on sympathetic activity and baroreflex sensitivity control to induce an anti-hypertensive action [42,43]. Other members of BPPs, with common hypotensive effect, have been identified in different species of snake venom including the golden lancehead viper (*Bothrops insularis*) [44], Southern American bushmaster (*Lachesis muta*) (Lm-BPP 1, Lm-BPP 2, Lm-BPP 3, Lm-BPP 4, and Lm-BPP 5) [45], the Mexican ground pit viper *Agkistrodon bilineatus* [46], *Lachesis muta rhombeata* (LmrBPP9) [47], and *Crotalus durissus cascavella* (BPP-Cdc) [48].

### 2.4. Cysteine-Rich Secretory Proteins 

Snake venom cysteine-rich secretory proteins (svCRISPs) are members of the CAP protein superfamily consisting of cysteine-rich secretory proteins (CRISPs), antigen 5 (Ag5), and pathogenesis-related 1 (PR-1) proteins, and they are part of the non-enzymatic protein group in snake venom characterized with a single polypeptide protein with molecular weight between 20 and 30 kDa [49].

Yamazaki et al. isolated a CRISP from the venom of the Japanese mamushi (*Gloydius blomhoffii*) designated ablomin. This protein has been shown to block high K^+^-induced contraction of arterial smooth muscle cells, in a similar way to calciseptine, a well-known blocker of L-type voltage-gated Ca^2+^ channels [50]. Two other ablomin homologous proteins characterized from the Japanese Habu (*Protobothrops flavoviridis*) and the Chinese sea snake (*Laticauda semifasciata*) venoms, termed triflin and latisemin respectively, also inhibited high K^+^-induced arterial contraction [51]. Another study reported the purification of a novel svCRISP from the venom of a Southern Pacific rattlesnake *Crotalus oreganus helleri*. The peptide, named Hellerin, induced a dramatic increase in blood vascular permeability in vivo and in vitro [52]. 

### 2.5. Disintegrins

Snake venom disintegrins are used as lead compounds for the development of drugs acting as antagonists of platelets integrins and are therefore pharmacologically well-defined as anti-platelet aggregation/antithrombotic drugs. Clinically, these drugs reduce the risk of acute ischemic events and prevent thrombotic complications. Disintegrins are non-enzymatic, relatively small (4–15 kDa), cysteine-rich proteins that are found in snake venoms, and their functional classification depends on their ability to interact with specific integrins, which is determined by the presence of a particular integrin-binding motif. Such motifs are localized in the hairpin loop and differ in the amino acid sequence (RGD, MLD, or R/KTS). The largest and most investigated family of disintegrins is the RGD-disintegrins [53,54]. Several disintegrins were isolated from snake venoms especially from viper venom and described as antithrombotic agents [55]. Trigramin, a disintegrin containing the RGD (Arg-Gly-Asp) sequence purified from the venom of the common bamboo viper (*Trimeresurus gramineus*), was shown to inhibit platelet aggregation in vitro and in vivo by blocking fibrinogen binding to aggregation agonist-stimulated platelets like fibrinogen binding ADP-activated platelets [56,57]. In human platelet-rich plasma (PRP), trigramin also inhibited the platelet aggregation induced by ADP, collagen, or epinephrine [57]. In another study, trigramin was reported to inhibit the formation of hemostatic plugs in mesenteric arteries of hamsters probably through the inhibition of fibrinogen and von Willebrand factor binding to the GPIIb/GPIIIa complex on the surface of the platelets [56]. Echistatin, a 49-residue protein, isolated from the venom of the saw-scaled viper (*Echis carinatus*), was shown to contain (i) the RGD-sequence, which is common to proteins that bind to the glycoprotein complex GPIIb/GPIIIa and (ii) the “proline-arginine-asparagine-proline” sequence, found in the A α chain of the human fibrinogen at position 267–270. A 10-residue segment of this protein shows 90% homology with a portion of trigramin sequence and that it inhibits fibrinogen-dependent platelet aggregation in a similar way to trigramin [58]. Barbourin, a novel disulfide-rich disintegrin from the venom of the ground rattlesnake (*Sistrurus miliarius barbourin*), is highly homologous to other snake venom disintegrins, except that it contains the KDG (Lys-Gly-Asp) rather than the RDG recognition motif [59]. These disintegrin peptides have been described as highly potent and selective GPIIb-IIIa antagonists that can be used as antiplatelet agents [60]. Many other disintegrins with common anti-platelet aggregation potential have been identified from snake venoms such as those of the Malayan pit viper *Calloselasma rhodostoma* [61], Halys pit viper (*Gloydius halys*) [62], and the fer-de-lance (*Bothrops asper*) [63].

### 2.6. Fibrinolytic Enzyme Activity

Snake venoms have also been shown to contain fibrinolytic enzymes, which could serve as templates for the development of alternative thrombolytic compounds of interest for clinical application since they should not be inactivated by serine protease inhibitors in the mammalian blood [64]. Fibrolase, a 23 kDa-zinc metalloproteinase, was originally isolated from the venom of the southern copperhead snake *Agkistrodon contortrix*. This fibrinolytic enzyme acts directly on fibrinogen/fibrin primarily cleaving α- and β-chains of fibrinogen but not the γ-chain [65]. Different studies showed that fibrolase can degrade fibrin clots in vitro and in vivo. Fibrolase is effective in digesting fibrin and human blood clots in a dose-dependent manner by acting directly on fibrin without activating plasminogen. On the other hand, using an animal model of arterial thrombosis, fibrolase dissolves femoral arterial clots after a single intravenous bolus administration. These data revealed the potential of this fibrinolytic enzyme to treat occlusive thrombotic disease, but the problem was that fibrolase is not a plasminogen activator [66]. Therefore, an alternative strategy is to produce a chimeric derivative from fibrolase that possesses the dual ability to degrade fibrin clots and to inhibit platelet aggregation and thrombus reformation. Since RGD-containing agents have been shown to inhibit rethrombosis following thrombus dissolution by plasminogen activators, a more effective fibrinolytic enzyme was engineered by covalently incorporating an RGD-like peptide into fibrolase. This chimera was shown to retain fibrinolytic activity and to inhibit platelet aggregation by binding to the fibrinogen receptor integrin αIIbβ3 on platelets, leading thrombus lysis and inhibition of rethrombosis [67]. Other fibrinolytic enzymes were identified and purified from other species of snake venom including lebetase from *Vipera lebetina* [68], basiliscus fibrases from the venom of the Mexican West-Coast rattlesnake (*Crotalus basiliscus basiliscus*) [67], and graminelysin I from *Trimeresurus gramineus* [68]. Besides isolated peptides, recombinant proteins were also produced for therapeutic purposes. As such, alfimeprase, a fibrolase derivative with thrombolytic activity, was developed for the treatment of stroke and catheter occlusion diseases [69].

### 2.7. Three-Finger Toxins

Three-finger toxins (3FTXs) are non-enzymatic proteins found in snake venoms especially Elapidae. These toxins contain 60–74 amino acid residues and 4–5 disulfide bridges. Their nomenclature derives from their structure formed by three β-stranded loops extending from a small, globular, hydrophobic core containing all four conserved disulfide bridges, resembling the three fingers of a hand [70]. These toxins have been shown to recognize a broad range of molecular targets like nicotinic and muscarinic acetylcholine receptors and L-type calcium channels, resulting in various biological properties [71]. Among 3FTXs, calciseptine and FS2 toxins were purified from the venom of the black mamba (*Dendroaspis polylepis polylepis*) and characterized as L-type calcium channel blockers that lead to a vasorelaxant effect on smooth muscles and to a hypotensive activity [72,73]. Muscarinic toxin α (MTα), another 3FTX from snake venom, has been shown to be a potent antagonist for the α2B adrenoreceptor, which can be used for the treatment of blood pressure disorders [74]. Another low molecular weight 3FTX, designated KT-6.9, was purified from monocled cobra (*Naja kaouthia*) venom and reported as an anti-platelet agent. This 3FTX was suggested to inhibit platelet aggregation by acting through ADP receptors located on the platelet surface and therefore can be used for the treatment of blood coagulation disorders [75].

## 3. Snake-Venom-Molecule-Based Drugs for Cardiovascular Disease

Snake venoms have been used in the pharmaceutical field for the discovery and development of drugs to treat several diseases including CVDs. Here, we describe different drugs that were developed based on snake venom compounds for the purpose of CVD treatments (Table 2). Some of these drugs have been approved for medical use and are available on the market, while others are still under clinical trials or have not reached an official approval.

### 3.1. Captopril

Initially, teprotide, a short peptide isolated from the venom of *Bothrops jaracaca*, was used as a molecular template for the design of the ACE inhibitors—namely captopril and enalapril. Captopril was the first active snake-venom-molecule-based drug, approved by the Food and Drug Administration (FDA) for its use in the treatment of hypertension, a risk factor for CVDs. The drug is derived from a bradykinin-potentiating peptide (BPP) isolated from the venom of the *Bothrops jararaca* snake, which potentiates in vivo and in vitro pharmacological actions of bradykinin (see Section 2.3). The ability of BPP to inhibit the proteolytic enzymes that inactivate bradykinin and catalyze the conversion of angiotensin I (Ang I) to angiotensin II (Ang II) made them promising templates for anti-hypertensive drug design [37,81,82]. Captopril, known as an angiotensin-converting enzyme (ACE) inhibitor, exerts its role on the renin-angiotensin-aldosterone system, which is a key regulator of blood pressure. Inhibiting ACE leads to two major effects that result in blood pressure decrease: (i) prevention of the hydrolysis of bradykinin that binds to its receptor (BK receptor) and induces an increase in NO production and vasodilation, and (ii) inhibition of Ang I to Ang II conversion, which is known to be a vasoconstrictor agent by binding to AT1 receptor [83,84]. Clinical studies reported that captopril induces a significant acute potentiation of endothelium-dependent vasodilation (EDV) in hypertensive patients associated with defects in EDV [85]. Besides being used to treat hypertension, captopril has been also used to treat several other diseases including renal disease in diabetics and heart failure after myocardial infarction [9,86].

### 3.2. Aggrastat 

Aggrastat (or tirofiban) is a drug derived from a snake venom disintegrin called echistatin, which is a synthetic non-peptide compound that mimics RGD (see Section 2.5). Tirofiban is a synthetic highly specific antagonist of glycoprotein IIb/IIIa, provoking platelet aggregation inhibition. The drug was approved by the FDA in 1988 for its use in the treatment of acute coronary ischemic syndrome and the prevention of thrombotic complications [87]. This drug reduced major adverse cardiac events in patients undergoing percutaneous coronary intervention and in those with acute coronary syndromes when administrated intravenously in combination with aspirin and heparin [88,89].

### 3.3. Integrilin

Integrilin (or eptifibatide) was derived from a KGD-containing protein purified from the venom of the Florida ground rattlesnake. This synthetic cyclic heptapeptide, with six amino acids and one mercaptopropionyl residue, exerts its role by binding to the glycoprotein IIb/IIIa of human platelets, preventing the binding of fibrinogen and then preventing platelet aggregation and thrombus formation. In 1988, the drug was approved by the FDA for the treatment of acute coronary disease and for anti-thrombotic therapy [95,96,97].

### 3.4. Defibrase

Batroxobin is a serine-proteinase isolated from the venom of the Brazilian lancehead snake (*Bothrops moojeni*) and the common lancehead snake (*Bothrops atrox*) with thrombin-like activity [98]. It converts fibrinogen into fibrin, through the cleavage of the α chain [99]. It exhibits anticoagulation and plasma fibrinogen degradation properties, without interfering with the function of the platelets. It has been approved in several countries for its defibrinogenating activity, in various conditions including acute cerebral infarction and pulmonary embolism [94].

### 3.5. Alfimeprase 

Alfimeprase is a recombinant protein derived from the fibrinolytic enzyme (fibrolase) isolated from of the southern copperhead snake (Agkistrodon contortrix contortrix) [100]. Alfimeprase has a potent fibrinolytic activity and was successful in the treatment of patients with acute peripheral arterial occlusion [101]. However, the drug is not approved by the FDA and has reached phase III of clinical trials, but it failed to meet the expected end points. Nuvelo discontinued the clinical development of alfimeprase, and the program was dropped in March 2008 [102,103,104].

### 3.6. Viprinex 

Viprinex, also known as Ancrod, is a serine protease of 258 residues that was purified from Malayan pit viper venom. Viprinex has been reported as a defibrinogenating agent for the treatment of acute ischemic stroke, by blocking additional clot formation, as well as other diseases including heparin-induced thrombosis and thrombocytopenia. This drug reached phase III of clinical trials [105,106,107] but failed to get a clinical approval, since it was found to have no therapeutic benefit in large scale stroke trial; this ended in the discontinuation of its administration [108,109].

## 4. Conclusions

CVDs are a leading cause of mortality worldwide, and researchers keep investigating new therapeutic strategies based on natural products to treat CVDs and manage their risk factors, such as hypertension. Due to their wealth in bioactive molecules, snake venoms are among the natural products that are mainly studied for the discovery, purification, and characterization of bioactive molecules that have potential in treating CVDs, among other diseases. The targets of some of these bioactive molecules and their mechanism of action is common to some extent, as they mainly prevent platelet aggregation. This helped in the characterization of molecules of interest and the development of some drugs that have been already approved for general use and are available in the market. The list will continue growing, as some drugs are currently in clinical trials, and many other could appear as studies continue dissecting snake venom composition and biological activities. 

## Figures and Tables

**Table 1:** Summary of snake venom components and their effects on the cardiovascular system.

Proteins and Peptides	Effects	References
**Enzymatic toxins**		
Phospholipase A2 (PLA2)	Hypotension	
	Vasorelaxation	[21,76]
	Anti-atherogenic activity	
Fibrinolytic enzymes	Fibrinolytic activity	[67,77]
	Inhibition of platelet aggregation	
**Non-enzymatic toxins**		
Natriuretic peptides (NPs)	Vasorelaxation (DNP; Coa-NP2)	
	Hypotension (Coa-NP2)	
	Increasing NO production (Coa-NP2)	[30,31]
	Cardioprotective action (Lebetin 2)	
Bradykinin-potentiating peptides (BPPs)	Hypotension	[40,42,48]
Cysteine-rich secretory proteins (CRISPs)	Inhibition of high K^+^-induced contraction	[51,78]
Disintegrins	Inhibition of platelet aggregation	[79,80]
Three-finger toxins (3FTXs)	Hypotension and vasorelaxation	[73,75]
	Inhibition of platelet aggregation	

**Table 2:** Approved snake-venom-based drugs for CVD treatment.

Snake Venom	Natural Peptide/Protein	Drug	Mode of Action	Indications	References
*Bothrops jararaca*	Bradykinin-potentiating peptide (BPP)	Captopril/ Enalapril	ACE inhibitors	Hypertension, renal disease in diabetics and heart failure after myocardial infarction	[85,90,91]
*Echis carinatus*	Echistatin	Aggrastat/ Tirofiban	GP IIb/IIIa antagonist	Acute coronary ischemic and prevention of thrombotic complications	[58,92]
*Sistrurus m. barbouri*	Barbourin	Integrilin/ Eptifibatide	GP IIb/IIIa antagonist	Acute coronary disease and anti-thrombotic therapy	[59,80]
*Bothrops moojeni* *Bothrops atrox*	Batroxobin	Defibrase	Cleavage of fibrinogen Aα subunit	Acute cerebral infarction and unspecific angina pectoris (+ sudden deafness)	[93,94]

## Data Availability

Not applicable.
